# Changes in Gut Microbiota in Rats Fed a High Fat Diet Correlate with Obesity-Associated Metabolic Parameters

**DOI:** 10.1371/journal.pone.0126931

**Published:** 2015-05-18

**Authors:** Virginie Lecomte, Nadeem O. Kaakoush, Christopher A. Maloney, Mukesh Raipuria, Karina D. Huinao, Hazel M. Mitchell, Margaret J. Morris

**Affiliations:** 1 School of Medical Sciences, UNSW Australia, Sydney, New South Wales, Australia; 2 School of Biotechnology and Biomolecular Sciences, UNSW Australia, Sydney, New South Wales, Australia; College of Tropical Agriculture and Human Resources, University of Hawaii, UNITED STATES

## Abstract

The gut microbiota is emerging as a new factor in the development of obesity. Many studies have described changes in microbiota composition in response to obesity and high fat diet (HFD) at the phylum level. In this study we used 16s RNA high throughput sequencing on faecal samples from rats chronically fed HFD or control chow (n = 10 per group, 16 weeks) to investigate changes in gut microbiota composition at the species level. 53.17% dissimilarity between groups was observed at the species level. *Lactobacillus intestinalis* dominated the microbiota in rats under the chow diet. However this species was considerably less abundant in rats fed HFD (P<0.0001), this being compensated by an increase in abundance of propionate/acetate producing species. To further understand the influence of these species on the development of the obese phenotype, we correlated their abundance with metabolic parameters associated with obesity. Of the taxa contributing the most to dissimilarity between groups, 10 presented significant correlations with at least one of the tested parameters, three of them correlated positively with all metabolic parameters: *Phascolarctobacterium*, *Proteus mirabilis* and *Veillonellaceae*, all propionate/acetate producers. *Lactobacillus intestinalis* was the only species whose abundance was negatively correlated with change in body weight and fat mass. This species decreased drastically in response to HFD, favouring propionate/acetate producing bacterial species whose abundance was strongly correlated with adiposity and deterioration of metabolic factors. Our observations suggest that these species may play a key role in the development of obesity in response to a HFD.

## Introduction

Obesity is now recognized as a worldwide epidemic, with its prevalence consistently increasing in most countries [1]. Multiple environmental and genetic factors are at play in the development of metabolic diseases. The last decade has seen the emergence of a new player thought to be involved in the onset of the metabolic syndrome associated with obesity: the gut microbiota. For example gut microbiota abundance and activity has been linked with several conditions that associate with metabolic syndrome including type 2 diabetes, non-alcoholic fatty liver disease, cardiovascular disease and obesity [2–4].

The gut microbiota is estimated to comprise over 10^14^ bacteria from more than 1000 different species. The results of the Human Microbiome Project by the National Institutes of Health described more than 70 bacterial phyla with four constituting the majority of mammalian intestinal microbiota (Bacteroidetes, Firmicutes, Actinobacteria, and Proteobacteria) and only two predominating in the intestinal tract: the Bacteroidetes and the Firmicutes[5]. Specific analyses of the adult rat gastrointestinal tract microbiota revealed the same four predominant phyla (Bacteroidetes, Firmicutes, Actinobacteria, and Proteobacteria) in caecal or faecal contents [6,7], making the rat therefore an ideal model to study influence of diseases on gut microbiota composition.

The first evidence of a change in gut microbiota composition in response to an obese phenotype was shown in genetic obese ob/ob mice; these mice displayed fewer Bacteroidetes and more Firmicutes [8]. Furthermore, the idea of an obesogenic gut microbial population emerged when the same authors discovered that the obesity phenotype can be transmitted by gut microbiota transplantation in mice [9].

Ley and colleagues confirmed these observations in human obese subjects [10], but the exact nature of the change in the gut microbiota phyla associated with obesity in humans remains controversial [11–14]. However, several studies have shown associations between bacterial richness and body mass index (BMI), adiposity, dyslipidemia and insulin resistance [15].

Gut microbiota composition is highly influenced by its host environment [16,17]. Diet is one of the various factors to which gut microbiota responds [18]. In animals, a high fat diet (HFD) results in altered abundance of the Bacteriodetes and Firmicutes phyla [8,19–23]. Changes in response to a HFD have also been reported at the class and order levels, but until recently technical limitations have prevented examination at a deeper level to enable identification of significant changes at the family or species level [24].

Understanding the complex host-microbiome cross-talk is essential to elaborate therapeutic strategies aiming to reverse deleterious changes caused by obesity and metabolic diseases. The human microbiome, which includes all micro-organisms that reside in or on the human body, contains 100 times more genes than the human genome. The microbiome interacts with the host in a multigenomic symbiosis contributing to essential physiologic functions such as immune-system modulation and energy metabolism. The gut microbiota influences the host metabolic phenotype via a range of mechanisms, one of them being the production of energetic substrates by fermentation, especially the short chain fatty acids, acetate, butyrate and propionate [25,26]. Acetate is the main SCFA in the colon and acts as a substrate for hepatic cholesterol synthesis and *de-novo* lipogenesis. The role of propionate is more controversial. It is a neoglucogenic susbstrate for the liver and has been showed to increase adipogenesis and inhibit lipolysis in mice adipose tissue. However, propionate may also counteract cholesterol synthesis and *de-novo* lipogenesis from acetate in the liver. Thus, the ratio of acetate/propionate plays a critical role in regulating lipid and cholesterol metabolism [26–28]. Butyrate is the main energy supply for the colonocytes and a lack of butyrate production could cause modifications in the structure of the intestinal epithelium leading to increased intestinal permeability and passage of molecules from the intestinal lumen to the bloodstream [29]. Among these molecules, endotoxins, lipopolyssacharides resulting from the degradation of the Gram negative outer membrane, are considered to play a key role in the development of the low grade inflammation associated with obesity [30,31]. Extensive research in animal models and humans demonstrate a relationship between fat feeding, gut leakiness, metabolic endotoxemia, obesity-associated chronic inflammation and onset of metabolic disease. Although this field is relevant to understanding the mechanisms linking gut microbiota and metabolic syndrome, in this study we adopted a different approach. By correlating changes in microbiota composition and an extensive suite of metabolic parameters, this study highlights bacterial species that are the most relevant to obesity-associated metabolic disease.

The gut microbiota is a potential therapeutic target for metabolic diseases. Although dietary interventions can normalise the composition of the gut microbiota in overweight and obese subjects, more targeted approaches are needed [32]. Two main strategies used to manipulate the intestinal microbial composition are selectively stimulating the growth and activity of certain species by administering either prebiotics or food supplements that contain living bacteria, probiotics [33,34]. However for these strategies to be efficient, they need to be targeted against specific species involved in the development of the metabolic syndrome. To date most studies that have analysed the gut microbiota associated with obesity have reported to the phylum level only. In the current study we used a 16S rRNA high throughput sequencing technology that has for the first time in rats, provided observations of changes in gut microbiota in response to a HFD at the species level. Furthermore we demonstrate that several changes in species abundance were tightly correlated with the deterioration of metabolic parameters associated with obesity, thus highlighting a role for these specific species in the development of obesity and possible therapeutic targets.

## Materials and Methods

### Ethics statement

This study was carried out in strict accordance with the recommendations of the Australian Code for the Care and Use of Animals for Scientific Purposes (1969), the Animal Research Act 1985 and New South Wales Animal Research Regulation 2010.

All animal procedures were approved by the University of New South Wales Animal Care & Ethics Committee (ACEC) (ACEC# 11/25A). All efforts were made to minimize animal suffering and stress. Euthanasia was performed under deep anaesthesia induced by a Ketamine/Xylazine mix.

### Animal care

6 week-old male Sprague Dawley rats from the Animal Research Centre (ARC, Perth, Australia) were housed in a clean facility, 2 per cage under a 12:12 h light/dark cycle. After acclimatisation, rats were split into two groups of equal average body weight (n = 10/10). Control rats were fed control chow (11 kJ/g, 12% fat, 21% protein, 65% carbohydrate as percent energy; Gordon’s Stockfeeds, NSW, Australia) whilst the HFD group was offered a choice of three different diets: the control chow, a commercial high fat pelleted diet SF03-020 (20 kJ/g, 43% fat, 17% protein, 40% carbohydrate; Specialty feeds, Glen Forest, WA, Australia) and a modified chow consisting of powdered chow, sweetened condensed milk and saturated animal fat (lard; 15.4 kJ/g; 51% fat, 10% protein, 38% carbohydrate) for 16 weeks. The average 24 hour food intake was calculated weekly by carefully collecting and weighing the food remaining in the cage and subtracting this from the known amount given.

### Glucose and Insulin Tolerance Test

A glucose tolerance test was performed at 21 weeks of age (13 weeks of diet) following an overnight fast. Two g of glucose/kg body weight (30% w/v) was administered intraperitoneally to each rat and blood glucose concentrations were measured at 0, 15, 30, 45, 60, 90, and 120 min using an Accu-check Go glucose meter (Roche diagnostic, Castle Hill, NSW, Australia). Blood samples for insulin measurement were collected in heparin coated tubes at 0, 15, 30, 60 and 120 min. Plasma samples obtained after blood centrifugation were stored at -20°C.

An insulin tolerance test was again performed at 22 weeks of age, 6 hours after food removal. One IU/Kg body weight of Insulin (100IU/mL Actrapid, NovoNordisk) was administered and blood glucose concentrations were measured at 0, 15, 30, 45, 60, 90, and 120 min.

### Sample collection

At 24 weeks of age, overnight fasted rats were tested for blood glucose then anaesthetised (xylazine/ketamine 15/100 mg/kg; Provet, Castle Hill, NSW, Australia). After measurement of body weight and naso-anal length, blood was collected in heparin coated tubes following cardiac puncture. Blood was centrifuged at 13,000 rpm for 5 min (Eppendorf Minispin; Crown Scientific, NSW, Australia) and the plasma stored at -20°C for hormone (leptin and insulin) and triglyceride measurements. Rats were killed by decapitation. Retroperitoneal and epidydimal white adipose tissues (RpWAT; epiWAT) were dissected and weighed. One faecal sample per animal was harvested from the terminal part of the caecum and stored at -80°C prior to examination.

### Plasma triglycerides, leptin and insulin assays

Plasma triglycerides were analysed colorimetrically (490 nm; iMark Microplate Absorbance Reader, Bio-Rad, Gladesville, NSW, Australia) using a commercially available triglyceride reagent (GPO-PAP, Roche diagnostic, Castle Hill, NSW, Australia) and glycerol standard (Sigma, Castle Hill, NSW, Australia). Plasma leptin and insulin concentrations were analysed using commercially available radioimmunoassay kits, according to manufacturer’s instructions (Merck Millipore, Billerica, MA, USA) and counted on a WIZARD^2^ Automatic Gamma Counter (PerkinElmer, Melbourne, VIC, Australia). Results were expressed as mean ± SEM. Anthropometric and metabolic parameters were analysed by Student’s two-tailed t-test after verification of the normality and data transformation when needed, using SPSS, version 20 (SPSS Inc., Chicago, USA).

### DNA extraction and microbial community sequencing

DNA extraction was performed using the ISOLATE Fecal DNA Kit (Bioline; Alexandria, NSW, Australia) according to the manufacturer’s instructions. The concentration and quality of DNA was measured using a Nanodrop ND-1000 Spectrophotometer (Nanodrop Technologies; Wilmington, USA).

The microbial community was assessed by high-throughput sequencing of the 16S rRNA gene. Tag-encoded FLX amplicon pyrosequencing (bTEFAP) was performed as described previously using the primers Gray28F (5’TTTGATCNTGGCTCAG) and Gray519r (5’ GTNTTACNGCGGCKGCTG) [35–38] with the primers numbered in relation to *E*. *coli* 16S rRNA. Generation of the sequencing library utilised a one-step PCR with a total of 30 cycles, a mixture of Hot Start and HotStar high fidelity taq polymerases, and amplicons originating and sequencing extending from the 28F with an average read length of 400 bp. The PCR was performed under the following conditions: 94°C for 3 min followed by 30 cycles of 94°C for 30 s; 60°C for 40 s and 72°C for 1 min; and a final elongation step at 72°C for 5 min. Tag-encoded FLX amplicon pyrosequencing analyses utilised a Roche 454 FLX instrument with Titanium reagents. This *bTEFAP* process was performed at the Molecular Research laboratory (MR DNA; Shallowater, TX) based upon established and validated protocols [35–38].

The Q25 sequence data derived from the high-throughput sequencing process was analysed employing a pipeline developed at Molecular Research LP. Sequences were first depleted of barcodes and primers, then short sequences <200 bp, sequences with ambiguous base calls, and sequences with homopolymer runs exceeding 6 bp were all removed. Sequences were then de-noised and chimeras were removed using the Black Box Chimera Check (B2C2) algorithm [39]. Operational taxonomic units (OTU) were defined after removal of singleton sequences with clustering set at 3% divergence (97% similarity) [40–46]. To determine the identity of bacteria, sequences were assembled into clusters and queried using a distributed BLASTn. NET algorithm [47] against a curated GreenGenes database. Database sequences were characterised as high quality based upon similar criteria utilised by RDP version 9 [48]. Using a. NET and C# analysis pipeline the resulting BLASTn outputs were compiled, validated using taxonomic distance methods, and data reduction analysis. Taxonomy was defined based on the following percentages: >97%, species; between 97% and 95%, unclassified species; between 95% and 90%, unclassified genus; between 90% and 85%, unclassified family; between 85% and 80%, unclassified order; between 80% and 77%, unclassified phylum; <77%, unclassified.

The raw sequence data are publicly available in European Nucleotide Archive (ENA), study accession number: PRJEB8565 (https://www.ebi.ac.uk/ena/submit/sra).

### Statistical analyses of microbial communities

Statistical analyses were conducted on both raw count data and data standardised to percent relative abundance. As both raw counts and standardised data yielded similar results, only % relative abundance results were presented to simplify the interpretation of data. The data was then square root transformed to further standardise the contribution of the taxa, in an attempt to address the dominating effect highly abundant taxa have on multivariate similarity measures. At all taxonomic levels, differences in microbial composition between diet groups were assessed using Permutational Multivariate Analysis of Variance (PERMANOVA) [49]. This procedure is a multivariate analogue of ANOVA except that pairwise distances/similarities between sampling units (in this case using the Bray-Curtis similarity coefficient) were used to calculate multivariate averages (centroids) and test statistics (pseudo-F). Probabilities were then obtained by comparing the pseudo-F value to a distribution of test statistics generated by random permutations of the data at hand. The PERMANOVA had two factors; diet type (fixed) and cage (nested in diet type). As PERMANOVA can be sensitive to differences in multivariate variance (dispersion), homogeneity of dispersions was checked using Permutational Analysis of Multivariate Dispersions (PERMDISP)[49]. This procedure was used to ascertain whether differences between diets were attributed to a location effect (differences in centroids but similar dispersions), a dispersion effect (similar centroids but differences in dispersions) or both. All statistical analyses were conducted on the statistical software packages PRIMER-E and PERMANOVA 6.

Similarity Percentages (SIMPER) analysis [50] was used to explore which species or taxonomic groups contributed to overall differences between diet types. SIMPER does not output probabilities, but calculates which taxa consistently differ between groups/treatments, and are thus most likely to be contributing to differences. Taxa with consistently greater average dissimilarity (δ_i_) between groups were considered to contribute most to differences between groups. Moreover, taxa with a larger ratio of average dissimilarity to its standard deviation (δ_i_/SD) indicate good discriminating taxa between groups. Cumulative % contribution of taxonomic identities and their corresponding δ_i_/SD were graphed, and taxa considered to contribute most to differences were determined by identifying where the % cumulative contribution levelled off. Upper limits of contributions displayed are 100% of average dissimilarity in phyla, up to 90% of average dissimilarity in class, order and family, and up to 50% of average dissimilarity in genus and species.

The diversity of each sample was analysed using the Shannon-Weaver diversity index for microbial composition at each taxonomic level. Student’s t-tests and Mann-Whitney U tests were conducted to compare diversity between diet types. Pairwise correlations were employed to detect associations between taxa and metabolic parameters.

## Results

### Effect of diet type on body weight and metabolic parameters

**Fig 1:**
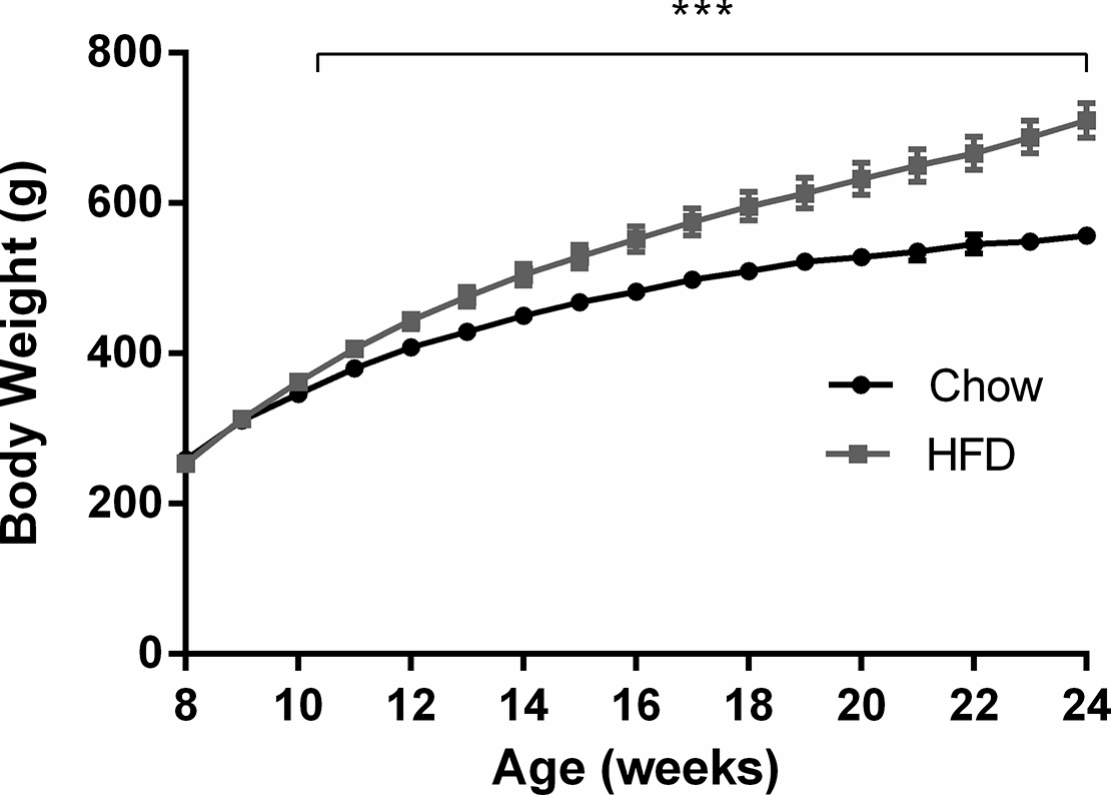
Body weight over the 16 week diet experiment. The black line represents the rats on chow diet (n = 10); the grey line represents the rats on high fat diet (n = 10). Results are expressed as mean ± SEM. ***P<0.0001, compared to control.

Body weights were standardised in all treatment groups prior to the commencement of the diet (Control: 242.9 ± 2.9 g; HFD: 241.7 ± 3.3 g). As expected, HFD fed rats gained more weight than controls, and three weeks after the commencement of diet, HFD fed rats were significantly heavier than chow fed animals (Chow: 355.4 ± 6.4 g; HFD: 375.9 ± 8.7 g; *P*<0.05; Fig 1). At the end point of the experiment (16 weeks of diet), HFD fed animals were 39% heavier than chow fed animals (*P* = 4.11E-7; Table 1) and consumed 25% more energy throughout the whole study compared to the chow fed group (total energy intake per rat Chow: 39450 ± 2439 kJ; HFD: 46968 ± 671 kJ with chow = 6.5%, commercial HFD = 82.4%, modified chow = 11.1% of total energy intake. n = 5 cages per group). As expected from the increased body weight, adipose tissue mass was markedly increased across both depots measured in the HFD fed group (*P*<0.001; Table 1).

**Table 1:** Metabolic parameters after 16 weeks of diet.

	Chow		HFD		
	Mean	SEM		Mean	SEM
Body Weight (g)	520.00	9.34		722.27 ***	25.94
rpWAT (g)	7.28	0.70		36.08 ***	4.29
epiWAT (g)	6.09	0.74		23.33 ***	1.81
Fat Mass (g)	13.37	1.37		59.41 ***	5.77
Glucose (mM)	4.7	0.20		5.1	0.30
Insulin (ng/ml)	0.64	0.14		0.89	0.17
Leptin (ng/ml)	4.79	0.74		18.44 ***	3.09
Triglycerides (mg/ml)	0.57	0.06		0.81 *	0.11

Chow = rats on chow diet (n = 10). HFD = rats on high fat diet (n = 10). RpWAT = retroperitoneal white adipose tissue. epiWAT = epidydimal white adipose tissue. Fat mass = sum of rpWAT and epiWAT. Results expressed as mean ± SEM.

*P<0.05

*** *P*<0.001, compared to control.

**Fig 2:**
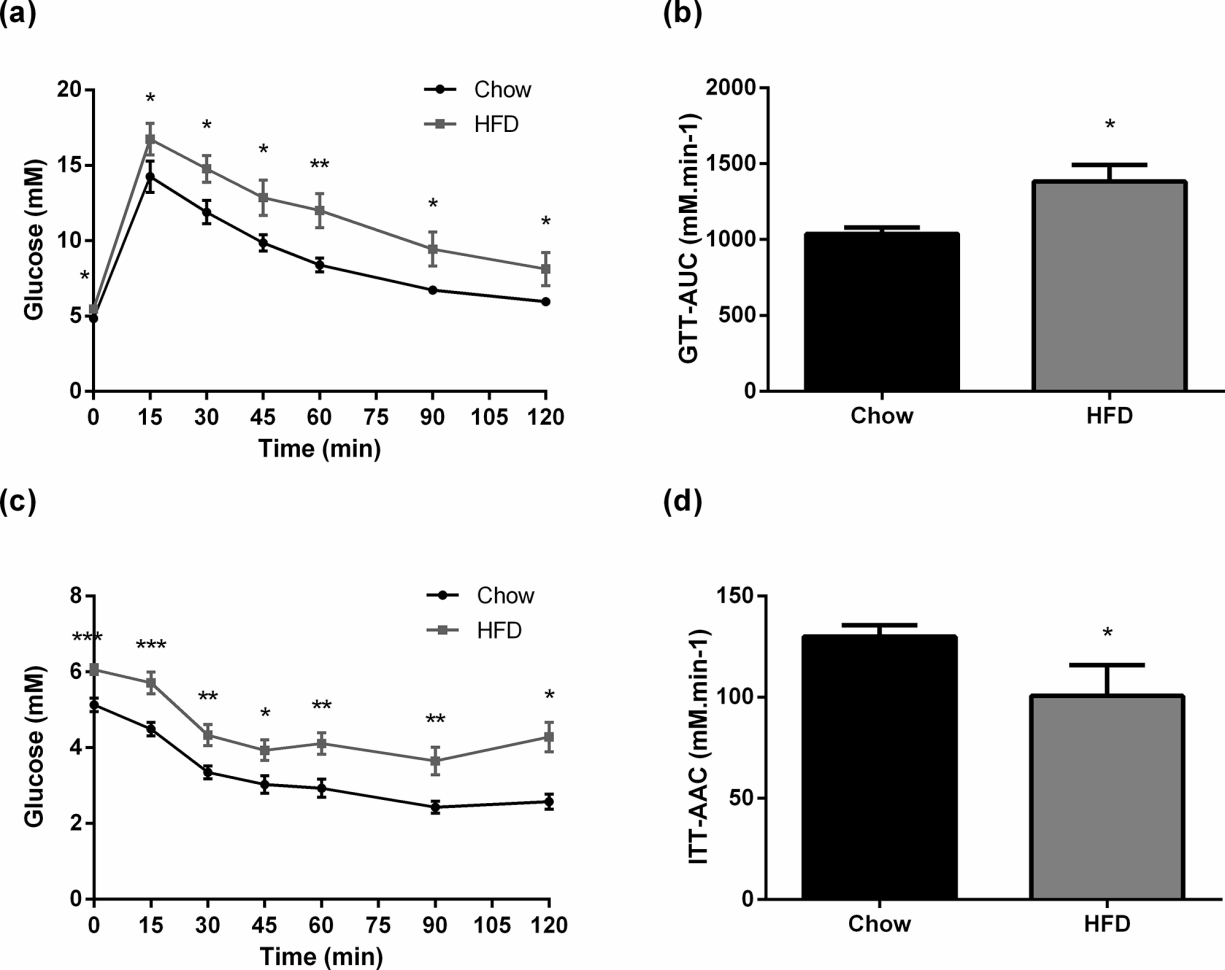
Glucose tolerance and insulin sensitivity. a) Glucose Tolerance test after 13 weeks of diet. The black line represents the rats on chow diet (n = 10); the grey line represents the rats on high fat diet (n = 10). b) Area Under Curve of GTT calculated at 90 min. The black box represents the rats on chow diet; the grey box represents the rats on high fat diet. c) Insulin Tolerance test after 14 weeks of diet. d) Area Above Curve for ITT calculated at 120 min. Results are expressed as mean ± SEM. *P<0.05; **P<0.005, compared to control.

In line with the increased fat mass, plasma leptin levels were four times higher in the HFD fed rats compared to control rats (*P*<0.001; Table 1). The plasma triglyceride concentration was also higher in the HFD group (*P*<0.05). Although the fasting insulin and fasting blood glucose concentrations were not significantly different between the two groups at the end of the study, the glucose tolerance test conducted at week 21 showed that the HFD fed rats were clearly glucose intolerant, with increased blood glucose concentrations across the 120 min test, yielding a significantly greater area under the curve (Fig 2A and 2B; AUC glucose at 120 min 1036.8 ± 42.5 mM/min and 1382.2 ± 110.3 mM/min, respectively; *P*<0.005). Insulin secretion in response to the glucose challenge was greater in the HFD group (Insulin 15 min after glucose injection Chow vs. HFD 2.60 ng/ml vs 3.72 ng/mL; *p*<0.05). An insulin tolerance test conducted at week 22 showed a higher fasting glucose concentration in the HFD group. HFD fed rats showed a similar reduction in glucose in the first hour post insulin injection (Fig 2C), but a decreased area above the curve (AAC) calculated at 120 min indicated reduced insulin sensitivity of the HFD fed rats (Fig 2D; AAC glucose at 120 min 129.9 ± 5.6 mM/min and 100.7 ± 15.1 mM/min, respectively; *P*<0.05).

### Effect of diet type on microbial composition

The gastrointestinal microbiota of the 24-week old rats fed chow (*n* = 10) or a HFD (*n* = 10) was analyzed using high throughput sequencing (average number of reads ± SEM: 10654 ± 516). The relative abundance of bacteria (S1 File) was compared across diet types with a nested PERMANOVA using the Bray Curtis similarity measure to construct distance matrices. The nested PERMANOVA had two factors: diet type (fixed) and cage (nested in diet type).

PERMANOVA analysis revealed significant differences in microbial communities between diet type across all taxonomic levels (phyla, F_1,8_ = 6.605, *P* = 0.017; class, F_1,8_ = 8.384, *P* = 0.006; order F_1,8_ = 8.466, *P* = 0.005; family, F_1,8_ = 9.671, *P* = 0.003; genera, F_1,8_ = 7.453, *P* = 0.006; species, F_1,8_ = 7.354, *P* = 0.003). Given that rats are coprophagic animals, the influence of shared caging on gut microbiota was tested. Variations associated with caging animals were not significant across all taxonomic levels (phyla, F_8,10_ = 0.738, *P* = 0.760; class, F_8,10_ = 1.021, *P* = 0.456; order, F_8,10_ = 1.020, *P* = 0.552; family, F_8,10_ = 1.095, *P* = 0.441; genus, F_8,10_ = 1.174, *P* = 0.305; species, F_8,10_ = 1.214, *P* = 0.249).

Variation of Bray-Curtis similarities were similar between treatments at all taxonomic levels (PERMDISP: phyla, F_1,18_ = 0.002, *P* = 0.961; class, F_1,18_ = 0.570, *P* = 0.534; order, F_1,18_ = 0.795, *P* = 0.504; family, F_1,18_ = 1.357, *P* = 0.320; genus, F_1,18_ = 0.276, *P* = 0.655; species, F_1,18_ = 1.009, *P* = 0.413).

### Effect of diet type on microbial diversity

**Fig 3:**
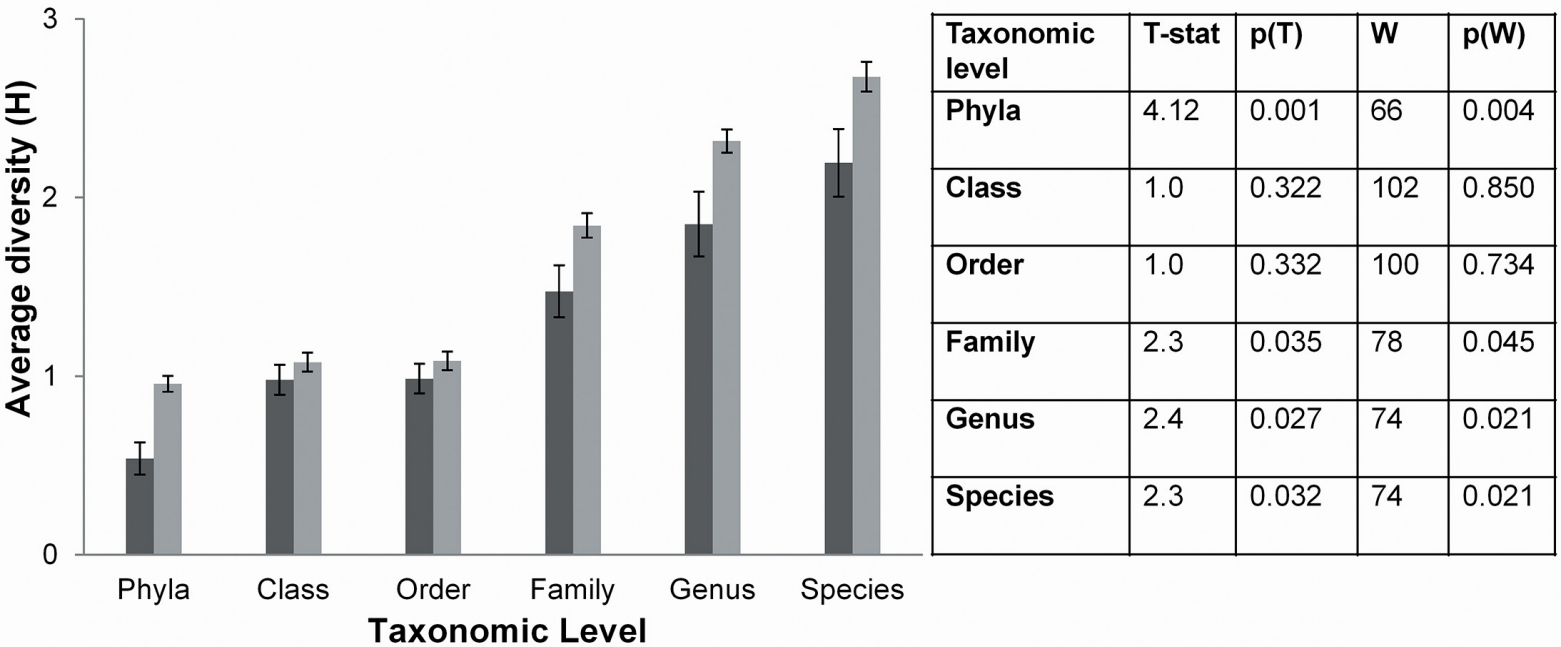
Average diversity between diet types using the Shannon-Weaver diversity index (H) for all taxonomic levels. Diversity statistics: T-test: df = 13 for phyla (unequal variance) and df = 18 for all other taxonomic levels; Manny-Whitney U test: df = 1. p(t) is *P*-value of the *t*-test, p(W) is *P*-value of the Mann-Whitney U test. Error bars display the standard error of mean.

The effect of diet type on microbial diversity within the gastrointestinal tract was determined using the Shannon-Weaver diversity index for microbial composition at each taxonomic level. Chow fed rats were significantly lower in diversity at the phyla, family, genus and species levels, whereas differences in diversity did not reach significance for the class and order taxa (Fig 3).

### Analysis of the changes in microbial communities

A preliminary Principle Component Analysis (PCA) was conducted to visualise differences in bacterial phyla composition between diet types, and to determine which phyla were most strongly associated with the differences observed. PCA confirmed that samples from rats fed chow and HFD formed distinct clusters in the ordination plot (S1 Fig). This separation was most apparent along the PC1 axis which explained 71.0% of the overall variation, and for which Bacteroidetes and Firmicutes had the highest correlation (0.406 and -0.836, respectively) (S1 Fig). The PC2 axis explained 22.5% of the overall variation (S1 Fig), however, no distinctions between diet groups were made through this component.

**Fig 4:**
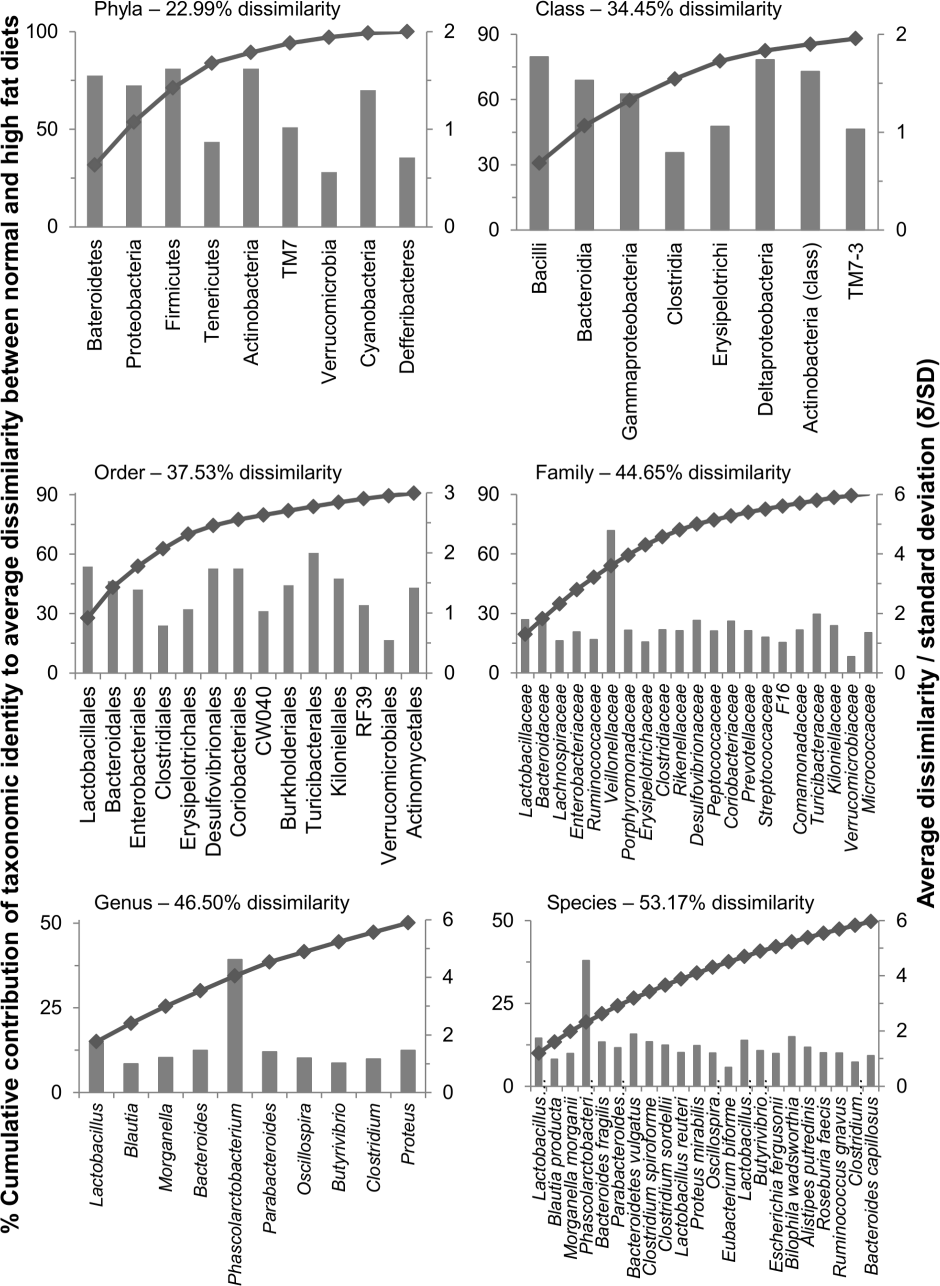
Taxa with the highest contribution to differences between the diet types identified through SIMPER analysis. The primary axis displays the cumulative contribution that each taxonomic identity contributes to average dissimilarity between diet types. Upper limits of contributions displayed are 100% of average dissimilarity in phyla, up to 90% of average dissimilarity in class, order and family, and up to 50% of average dissimilarity in genus and species. The secondary axis displays the average dissimilarity/standard deviation (δ/SD) of each taxonomic identity displayed. The % dissimilarity in microbial composition between diet types is shown in each graph.

SIMPER analyses across all taxonomic levels were employed to identify taxa with the highest contribution to differences between the diet types. The distinction between diet types were more apparent as the taxonomic level became more specific where SIMPER detected 22.9% dissimilarity at the phylum level which increased to 53.17% dissimilarity at the species level. At the phylum level, the cumulative contribution dissimilarity began to level off at the taxon Tenericutes, thus, Bacteroidetes, Proteobacteria, Firmicutes and Tenericutes contributed the most to differences between chow and high fat chow diets (Fig 4). Bacteroidetes, Proteobacteria and Firmicutes were shown to be better discriminators between diet types as their δ_i_/SD values were higher (Fig 4). At the class level, dissimilarity between microbial communities was calculated at 35.45% between the diet groups. The cumulative contribution to dissimilarity began to level off at Deltaproteobacteria. Bacilli (Bacillales and Lactobacillales) contributed most to differences between diet types followed by Bacteroidia, Gammaproteobacteria, Clostridia, Erysipelotrichi and Deltaproteobacteria (Fig 4). Of these taxa, Bacilli, Bacteroidia, Gammaproteobacteria and Deltaproteobacteria had higher δ_i_/SD values, and were thus better discriminators between diet types (Fig 4). The chow fed rat samples were dominated by Bacilli and Clostridia, while samples from HFD fed rats had a markedly reduced abundance of Bacilli and higher abundances of Bacteroidia, Gammaproteobacteria, Clostridia, Erysipelotrichi and Deltaproteobacteria (Table 2).

**Table 2:** Classification of taxa considered to contribute most to dissimilarity between diet types.

Phylum	Class	Family	Species		Chow (%)	High fat (%)
Firmicutes					83.3 ± 4.2	62.1 ± 3.5
	Bacilli				33.2 ± 8.4	1.2 ± 0.5
		Lactobacillales			33.1 ± 8.4	1.1 ± 0.5
			*Lactobacillaceae*		33.0 ± 8.4	0.8 ± 0.4
				*Lactobacillus intestinalis*	29.7 ± 8.0	0.014 ± 0.008
	Clostridia				50.1 ± 6.1	60.9 ± 3.6
		Closdtridiales			50.1 ± 6.1	60.9 ± 3.6
			*Lachnospiraceae*		29.0 ± 5.8	37.4 ± 3.9
				*Blautia producta*	1.9 ± 0.4	11.0 ± 4.4
			*Clostridiaceae*		3.7 ± 0.92	1.7 ± 0.5
			*Ruminococcaceae*		12.4 ± 2.9	17.0 ± 3.0
	Negativicutes				-	-
		Selenomonadales			-	-
			*Acidaminococcaceae *		-	-
				*Phascolarctobacterium*	0	2.1 ± 0.3
			*Veillonellaceae*		0	2.1 ± 0.3
	Erysipelotrichi				0.44 ± 0.21	4.0 ± 2.1
		Erysipelotrichales			0.44 ± 0.21	4.0 ± 2.1
			*Erysipelotrichaceae*		0.44 ± 0.21	4.0 ± 2.1
Bacteroidetes					5.1 ± 2.2	17.2 ± 3.6
	Bacteroidia				5.1 ± 2.2	17.2 ± 3.6
		Bacteriodales			5.1 ± 2.2	17.2 ± 3.6
			*Bacteroidaceae*		0.12 ± 0.07	5.5 ± 1.5
				*Bacteroides vulgatus*	0.035 ± 0.029	1.8 ± 0.4
				*Bacteroides fragilis*	0	2.4 ± 0.9
			*Porphyromonadaceae*		0.063 ± 0.033	3.0 ± 1.0
				*Parabacteroides distasonis*	0.055 ± 0.028	2.7 ± 0.9
			*Rikenelleaceae*		0.78 ± 0.42	1.61 ± 0.46
Proteobacteria					9.8 ± 2.8	15.1 ± 3.7
	γ-Proteobacteria				-	-
		Enterobacteriales			9.33 ± 2.79	13.62 ± 3.86
			*Enterbacteriaceae*		9.33 ± 2.79	13.62 ± 3.86
				*Morganella morganii*	4.8 ± 1.9	9.0 ± 3.4
	δ-Proteobacteria				0.014 ± 0.006	0.77 ± 0.23
		Desulfovibrionales			0.014 ± 0.006	0.77 ± 0.23
			*Desulfovibrionaceae*		0.014 ± 0.006	0.77 ± 0.23
				*Bilophila wadsworthia*	0.010 ± 0.004	0.75 ± 0.23
Tenericutes					0.75 ± 0.19	4.4 ± 2.3

Determined using SIMPER graphs where % cumulative contribution began to level off. Average % relative abundances and standard errors of the means are shown.

Microbial communities showed 37.53% dissimilarity at the order level. Similar to the class level, the % cumulative contribution began to level off at Desulfovibrionales, an order within the Deltaproteobacteria. Lactobacillales and Clostridiales dominated chow fed rat samples, whereas the HFD was associated with a markedly lower abundance of Lactobacillales and a higher abundance of Clostridiales, Bacteroidales, Enterobacteriales, Erysipelotrichales and Desulfovibrionales (Fig 4 and Table 2). This pattern was consistent with class level bacterial composition in which these orders represented ~100% of their corresponding class level abundance (Table 2). Of these taxa, Lactobacillales, Bacteroidales, Enterobacteriales and Desulfovibrionales had the highest δ_i_/SD, and were thus better discriminators between diet types (Fig 4).

The dissimilarity increased again to 44.65% at the family level, with the % cumulative contribution beginning to level off at the taxon *Erysipelotrichaceae* (Fig 4). As expected, *Lactobacillaceae* dominated the microbiota within rats under chow diet, a finding that is likely to be associated with the lower diversity of the microbiota in these rats when compared to rats fed HFD (Fig 3). This is supported by the considerably lower abundance of *Lactobacillaceae* in rats fed HFD (Table 2), and a greater abundance of other important groups including *Bacteroidaceae*, *Lachnospiraceae*, *Enterobacteriaceae*, *Ruminococcaceae*, *Veillonellaceae*, *Porphyromonadaceae* and *Erysipelotrichaceae*. Of these taxa, *Veillonellaceae* had the highest δ_i_/SD value, and was likely to be the best discriminator between diet types at this taxonomic level, most probably due to its absence in chow fed rats.

**Fig 5:**
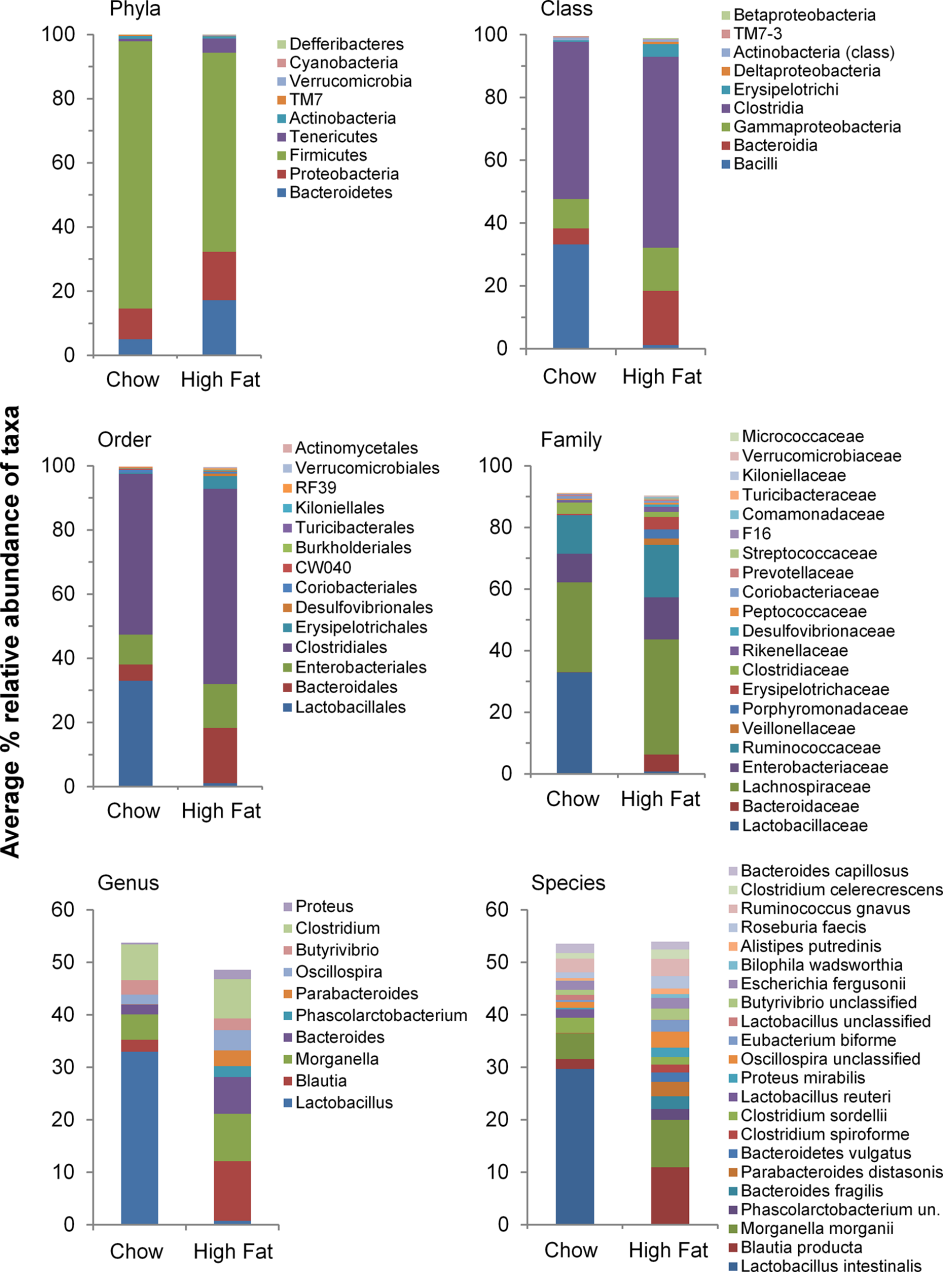
Average composition of taxa between rats consuming chow and high fat diets. These taxa contribute to 100% of average dissimilarity in phyla, up to 90% of average dissimilarity in class, order and family, and up to 50% of average dissimilarity in genus and species as shown in SIMPER graphs (Fig 4).

The dissimilarities of microbial communities at the genus and species levels were 46.5% and 53.71%, respectively. The % cumulative contribution began to level off at *Parabacteroides* and *Bacteroidetes vulgatus* for the genus and species, respectively (Fig 4 and S2 Fig). Again, *Lactobacillus*, and more specifically *Lactobacillus intestinalis* dominated the microbiota in chow fed rats (Fig 4 and Table 2). In contrast, *Lactobacillus intestinalis* were considerably lower in rats fed a high fat diet, with greater abundance of other important genera including *Blautia*, *Morganella*, *Bacteroides*, *Phascolarctobacterium* and *Parabacteroides* (Table 2). Within these genera, the species of interest were *Blautia producta*, *Morganella morgani*, *Phascolarctobacterium* unclassified, *Bacteroides fragilis*, *Parabacteroides distasonis*, and *Bacteroides vulgatus* (Table 2). Of these taxa, *Phascolarctobacterium* had the highest δ_i_/SD value, again most probably due to its absence in chow fed rats (Table 2). Overall, distinct community profiles were observed between diet types at all taxonomical levels, and a significantly higher diversity was observed in HFD fed rats at phylum, family, genus and species level (Fig 5).

### Correlation of microbial abundance with metabolic parameters

**Fig 6:**
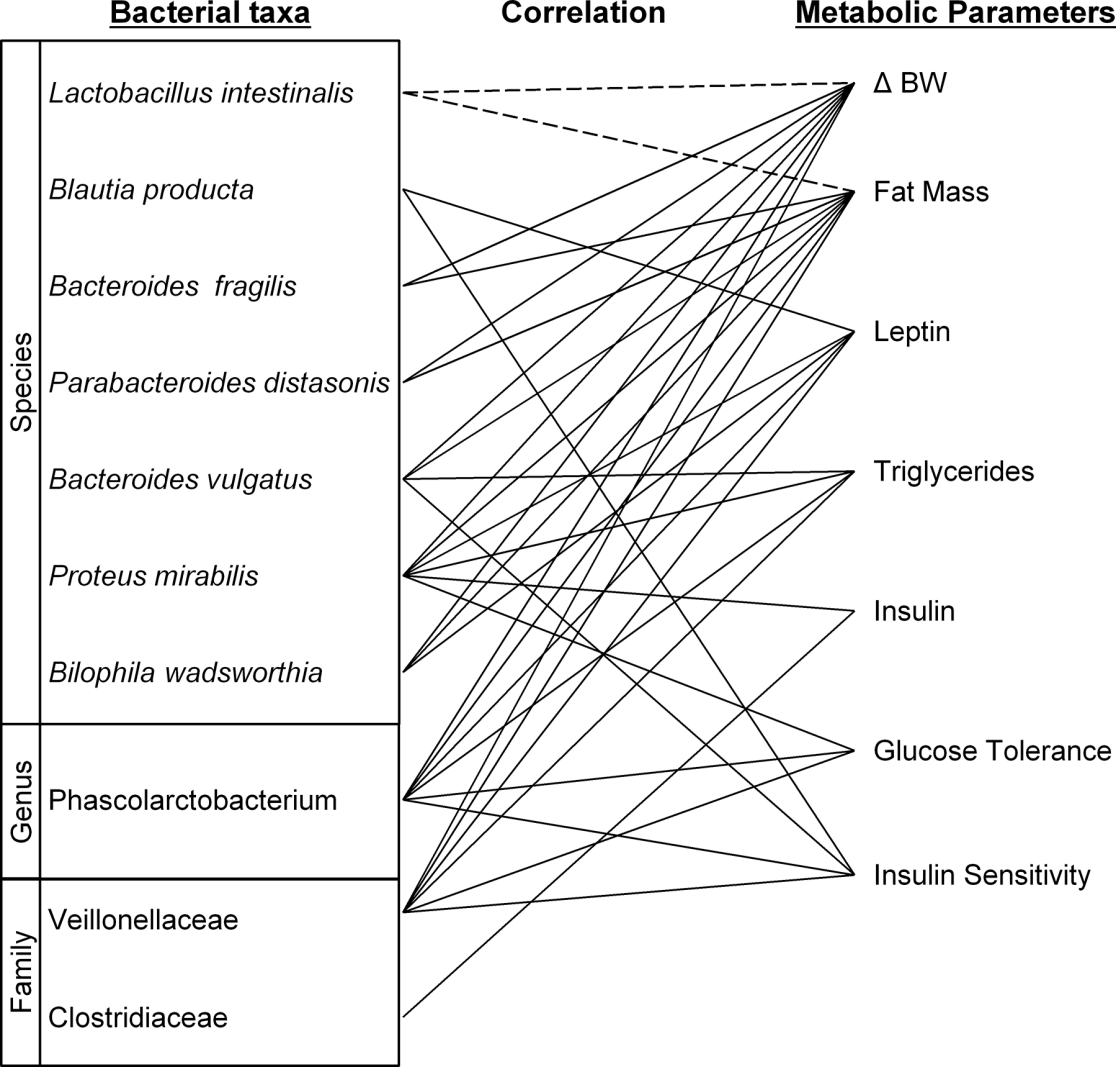
Correlation of abundance of bacterial taxa and metabolic parameters associated with obesity. Correlation between abundance of taxa identified to be contributing most to dissimilarity between diet types and metabolic parameters are represented by solid line for positive correlations and interrupted line for negative correlations. Only significant correlations, P value < 0.05, are represented. Metabolic parameters represented are: body weight gain (ΔBW), fat mass estimated by the cumulative weight of retroperitoneal and epidydimal fat, plasma leptin, insulin and triglyceride concentration, glucose tolerance estimated by the area under curve at 90 min of glucose tolerance test, and insulin sensitivity estimated by area above curve at 120 min of insulin tolerance test as shown in Fig 2.

Correlations of bacterial abundance with measurements of seven metabolic parameters related to obesity were performed on all rats for taxa contributing most to the dissimilarity between diet groups (Fig 6). The parameters tested were: Change in body weight, fat mass at killing, leptin, insulin and triglyceride plasma concentrations, glucose tolerance expressed by AUC GTT and insulin sensitivity expressed by AAC ITT.

The abundance of both the taxa *Veillonellaceae* and *Phascolarctobacterium* correlated with most metabolic parameters, a positive correlation being observed with six of the seven parameters, with no correlation being shown with plasma insulin concentration.

The abundance of *Bacteroides fragilis*, *Bacteroides vulgatus*, *Parabacteroides distasonis* and *Bilophila wadsworthia* were all positively correlated with both change in body weight and fat mass. *Bacteroides vulgatus* was also correlated with triglyceridemia and insulin sensitivity, while *Bilophila wadsworthia* correlated also with plasma leptin concentration.

*Lactobacillus intestinalis* was the only species whose abundance displayed a negative correlation with two major parameters: change in body weight and fat mass.

Of interest, while the % relative abundance of *Proteus mirabilis* differed between rats on chow and on HFD (chow: 0.29 ± 0.16; high fat: 1.8 ± 0.5), this taxon was not identified as a major contributor to dissimilarity between diet types using SIMPER. However, this species was significantly correlated with delta body weight (*r* = 0.632; *P* = 0.0028), triglyceride levels (*r* = 0.532; *P* = 0.023), leptin (*r* = 0.569; *P* = 0.0088) and insulin (*r* = 0.664; *P* = 0.0014) concentrations, indicating it may indeed be an important contributor. In addition, Actinobacteria (*r* = 0.502; *P* = 0.024), and more specifically *Eggerthella lenta* (*r* = 0.565; *P* = 0.0094), were correlated with insulin concentration of the rats.

## Discussion

In response to chronic HFD consumption, we observed a decrease in Firmicutes phylum, which was largely accounted for by a reduced abundance of *Lactobacillus* species. The abundance of this species was negatively correlated with fat mass and body weight, stressing the role of lactobacilli in metabolic syndrome development. Moreover, these correlations between metabolic parameters and abundance of microbial species point to the involvement of propionate and acetate-producing bacteria in the response to HFD. While a lot of studies have analysed gut microbiota at the phylum level, due to the progress of 16S rRNA high-throughput sequencing, in the current study we describe the microbiota composition in our rat model of diet-induced obesity down to the family, genus and species level. However, despite the high level of accuracy in current methods used for taxonomy assignment, the coverage offered by massive sequencing methods, with short read lengths, may be too low to solve accurately taxonomy classification at the species level.

To date many studies have reported the influence of obesity on the level of specific phyla, with changes in the ratio of Firmicutes to Bacteroidetes being reported both in humans and rodents. The relative proportion of Bacteroidetes has been reported to be decreased in obese as compared with lean people [10]. Increased abundance of Firmicutes in the intestinal microbiota of obese patients has been suggested to increase the capacity to harvest energy from the diet, thus promoting more efficient absorption of calories and subsequent weight gain[9]. Furthermore, a greater proportion of Firmicutes to Bacteroidetes has been described in genetically or diet-induced obese mice [8,22,51] and rats fed a HFD [21,23,52] relative to controls.

Based on the sequence analysis undertaken in this study, it would appear that the involvement of these phyla differs in our model. The consumption of a HFD resulted in a relative decrease in the abundance of Firmicutes and an increase in the abundance of Bacteroidetes. One study by Schwiertz *et al*. reported similar results in overweight and obese human subjects, with a shift in the ratio of Firmicutes to Bacteroidetes in favour of Bacteroidetes [13].

We reported an increase in microbial diversity in faeces from our HFD fed rats. This differs from several human studies, where no change or decreased gut microbial diversity was associated with obese phenotype [15,32,51]. However, increased bacterial diversity has been reported in obese compared to non-obese type 2 diabetic patients [53]. In this study, Larsen et *al*. compared gut microbiota composition of type 2 diabetic patients and healthy volunteers; a decrease in Firmicutes phylum in individuals with diabetes and a positive correlation of Bacteroidetes to Firmicutes ratio with plasma glucose was observed [53]. This “diabetic” microbiota profile is similar to our finding in chronically HFD fed rats, which presented both glucose-intolerance and insulin-resistance, two features of type 2 diabetes. Moreover, the perceived increase in microbial diversity of our HFD rats may have resulted from the dramatic drop in the levels of L. intestinalis which dominated the microbiota of control rats. The phylogenic classification of the Mollicutes class could also explain some of the differences between our model and the literature. Originally classified in the Tenericutes phylum, based on their dissimilarity with the classic Gram-positive bacteria, the phylogeny of Mollicutes has been questioned recently by several studies, as 16S rRNA sequence analysis reveal features similar to the Firmicutes [54]. In our analysis, the Mollicutes were considered under the Tenericutes phylum, which is increased in our HFD fed rats. This is in line with the results of a study by Turnbaugh *et al* that showed a bloom in Mollicutes in response to diet-induced obesity in mice. These authors classified the Mollicutes as Firmicutes and described them as the major contributor of the increased Firmicutes/Bacteriodetes ratio [55].

Specific analyses of the adult rat gastrointestinal tract microbiota revealed that the richness of bacterial species in the rat intestine is of the same order of magnitude or even higher than in the human gut microbiota [56]. The same 4 predominant bacterial phyla described in humans (Bacteroidetes, Firmicutes, Actinobacteria, and Proteobacteria) are detected in rat cecal or fecal contents [6,7], suggesting rats are a good model to study the influence of diet on gut microbiota. Nevertheless, given that major differences exist in the gut microbiota of human and rats at the species level, caution needs to be taken in the translation of findings from the rat model to humans. For example, in contrast to humans [57], *Lactobacillus* species represent a significant proportion of the rat microbiota and can reach 10–15% of the total sequence reads [6,7,58].

The global decrease in Firmicutes observed in rats fed a HFD was shown to be mainly due to a dramatic drop in the abundance of *Lactobacillus intestinalis*. The level of this bacterium was negatively correlated with the change in body weight and leptin levels, providing further evidence that its decrease in HFD fed rats is associated with the obese phenotype. Our finding is supported by a study by Zhao *et al*. showing that *Lactobacillus* spp. are decreased in the distal oesophagus of rats fed a HFD [59]. Moreover, Santacruz *et al*. [60] found significantly increased levels of *Lactobacillus* species in microbiota from overweight adolescents undertaking a lifestyle intervention to reduce obesity. Interestingly, long-term ingestion of *Lactobacillus* spp. has been found to decrease body weight in both rats [61] and humans [62] suggesting that *Lactobacillus* spp may be beneficial in metabolic disorders, and emphasising its importance for maintaining a healthy gastrointestinal tract. The beneficial health effects of lactobacilli are further supported by work correlating the abundance of lactobacilli in the gut with the lifespan of mice [63]. Furthermore, lactobacilli have been shown to be involved in maintaining intestinal barrier integrity through maintenance of cell-to-cell junctions and promotion of epithelial repair after injury. Thus, a decrease in their abundance is likely to increase bacterial endotoxin passage into the bloodstream, a major factor contributing to inflammation status and adiposity in obesity [30,31]. However, effects of *Lactobacillus* on obesity have been shown to be species and strain dependent and associated with either weight loss or weight gain [64,65].

Unlike the reduction in *Lactobacillus intestinalis*, a number of species from the Firmicutes phylum showed increased abundance in our obese rats, including the taxon *Erysipelotrichaceae*. Previous studies have shown these bacteria to be enriched in faecal samples from obese humans, genetically obese mice and high fat diet-associated mice, and to be closely linked to energy homeostasis and adiposity [66,67]. Moreover, our detected increase in the abundance of the Firmicutes family *Ruminococcaceae* and Bacteriodetes family *Rikenellaceae* and *Enterobacteriaceae* in rats fed a HFD is consistent with the findings of Kim *et al*. who found these taxa to be increased in mice consuming a HFD [68]. An increase in *Enterobacteriaceae* in diet-induced obese rats has also been reported by Barbier de la Serre *et al*. [21]. Increased abundance of *Enterobacteriaceae* has been associated with gut inflammation: induction of colitis in rodents has been reported to increase abundance of this family [69]. Finally, *B*. *fragilis* was detected in our HFD fed rats, but was absent in controls. Vael *et al* reported high intestinal concentrations of *B*. *fragilis* in infants aged between three weeks and one year to be associated with a higher risk of obesity later in life [70].

A major strength of this study was our ability to test the association between the gut microbiota composition and various metabolic parameters associated with obesity, and thus to determine bacterial species relevant to obesity. Responses to the HFD of some taxa were found to correlate more with body weight and fat mass and others more with glucose /insulin metabolism. Based on these findings, it can be hypothesised that species that correlate with changes in body weight and leptin, such as *Lactobacillus intestinalis* or *Bacteroides fragilis*, are more likely to be indirectly involved in increases in adiposity, while those correlating with plasma insulin level, GTT/ITT responses, such as the *Clostridiaceae* family, might be more associated with an insulin-resistance/glucose intolerance phenotype.

Our analysis highlights three taxa that correlated with six out of seven tested metabolic parameters: the *Veillonellaceae* family and *Phascolarctobacterium* species, both members of the same class within the Firmicutes phylum, and *Proteus mirabilis*, a species belonging to the Proteobacteria phylum. As these taxa are amongst those most contributing to dissimilarity between diet types, their strong relationships with the metabolic readouts implicates them in the response to a HFD and possibly the obesity and diabetes phenotype we observed.

*Proteus mirabilis* is part of the normal microbiota of the intestinal tract [71]. But an increased abundance of this bacterium has been associated with several inflammatory syndromes including inflammatory arthritis and rheumatoid arthritis and, even more interestingly, inflammatory bowel disease [72,73], suggesting a possible participation of *P*. *mirabilis* in the establishment of low grade inflammation associated with obesity.

*Phascolarctobacterium* spp. and *Veillonellaceae* produce high amounts of the short chain fatty acids (SCFA) acetate and propionate [74,75]. The majority of SCFA in the gut are derived from the fermentation by bacterial species of complex carbohydrates present in intestinal content such as dietary soluble fibres or resistant starch. The main SCFA present in the mammalian gut are acetate, propionate and butyrate. *Phascolarctobacterium* spp. specialise in the utilisation of succinate produced by other bacteria [75]. In parallel, the increased abundance of *Bacteroides* and *Parabacteroides*, both major producers of succinate [76], was positively correlated with body weight.

In our model, we observed a global decrease in butyrate-producing species, such as *Clostridiaceae*, in favour of acetate and propionate-producing species. Schwiertz *et al*., showed that the proportion of individual SCFAs was significantly change in favour of propionate (41%) over butyrate in both overweight (*P* = 0.019) and obese subjects (*P* = 0.028) [13]. Butyrate, produced by the gut microbiota, is the primary energy supply for colonocytes and is known to influence their structure and function. A decrease in butyrate content of the gut of germ-free mice has been shown to promote leakiness of the gut barrier [29,77], associated with increased endotoxemia and inflammation. Moreover, butyrate has been described to have anti-inflammatory properties which are associated with a reduction in leucocyte and macrophage recruitment and production of pro-inflammatory cytokines [78,79]. The decrease in butyrate-producing species and lactobacillus, species shown to protect the intestinal barrier integrity, could increase gut leakiness, favouring the development of metabolic endotoxemia and obesity-associated inflammation [29,30,31,77].

Most of the taxa whose abundance positively correlates with the metabolic parameters tested in our model (7 out of 14), are involved in acetate or propionate production (e.g. *Veillonellaceae*, *Phascolarctobacterium* spp., *Blautia producta*). In line with these results, Zhang *et al*. [63] reported significantly increased levels of acetic acid and propionic acid in rats fed a HFD. While butyrate is mainly used by colonocytes, acetate and propionate are largely taken up by the liver. Acetate is used as a substrate for cholesterol and fatty acid synthesis, thus promoting hypercholesterolemia, hypertriglyceridemia and the development of liver steatosis, as observed in our HFD fed rats. Propionate is a substrate for hepatic gluconeogenesis and has been reported to inhibit cholesterol synthesis in hepatic tissue. It has also been described to lower plasma lipids in humans [26]. However, these anti-lipogenesis effects of propionate are controversial. On the other hand, propionate has been shown to promote, through the activation of G protein-coupled receptor 43 or free fatty acid receptor 2, inhibition of lipolysis and adipocyte differentiation leading to increased adiposity [80,81].

## Conclusion and Perspectives

Several studies have shown altered composition of the gut microbiota in the context of obesity and type 2 diabetes in humans and rodents and have suggested that these changes could contribute to the onset of these metabolic diseases. In the current study, detailed analysis of gut microbiota composition and its correlation with metabolic measures in rats has highlighted several bacterial species that are strongly linked to the development of metabolic syndrome in response to a HFD. These species could represent interesting therapeutic targets, especially *Lactobacillus* species, which dramatically decreased in response to a HFD in our model and negatively correlated with gain in body weight and fat mass. Strategies to increase *Lactobacillus* species have shown anti-diabetic and anti-inflammatory effects in type 2 diabetes models, as well as weight loss in HFD fed mice [82–85]. Our model also underlines the correlation between obesity and decrease in butyrate-producing species. Intestinal butyrate production can be manipulated through consumption of dietary non-digestible carbohydrates such as oligofructose or amylase-resistant starch [33,86]. Using butyrate as a prebiotic, Gao et *al*. [87] demonstrated that supplementation of HFD with butyrate prevented and reversed insulin resistance in dietary-obese mice. These beneficial effects of pre- and probiotics on obesity and diabetes animal models are promising. However, caution needs to be exercised in the translation of these therapeutic strategies to humans as the relationship between changes in gut microbiota composition and metabolic effects are still not fully understood.

## Supporting Information

S1 Fig Principal components analysis of bacterial phyla between two different diet types. **a)** PC1 explained 71% of the variation, PC2 22.3% and PC3 6.5%. Star: chow diet; circle: high fat diet. **b)** Component loading derived from PCA analysis of bacterial phyla.
(TIF)

S2 Fig Contributions of taxa to differences between the diet types identified through SIMPER analysis. The primary axis displays the cumulative contribution that each taxonomic identity contributes to average dissimilarity between diet types. Upper limits of contributions displayed are 90% of average dissimilarity in genus and species. The secondary axis displays the average dissimilarity/standard deviation (δ/SD) of each taxonomic identity displayed.
(TIF)

S1 File Taxonomy analysis. Raw count data and data standardised to percent relative abundance per phylum, class, order, family, genus and species.
(XLSX)
