# Supplementary figures and images for: Changes in Gut Microbiota in Rats Fed a High Fat Diet Correlate with Obesity-Associated Metabolic Parameters
Source: PLoS One. 2015 May 18;10(5):e0126931. doi: 10.1371/journal.pone.0126931 (PMC4436290; doi:10.1371/journal.pone.0126931)

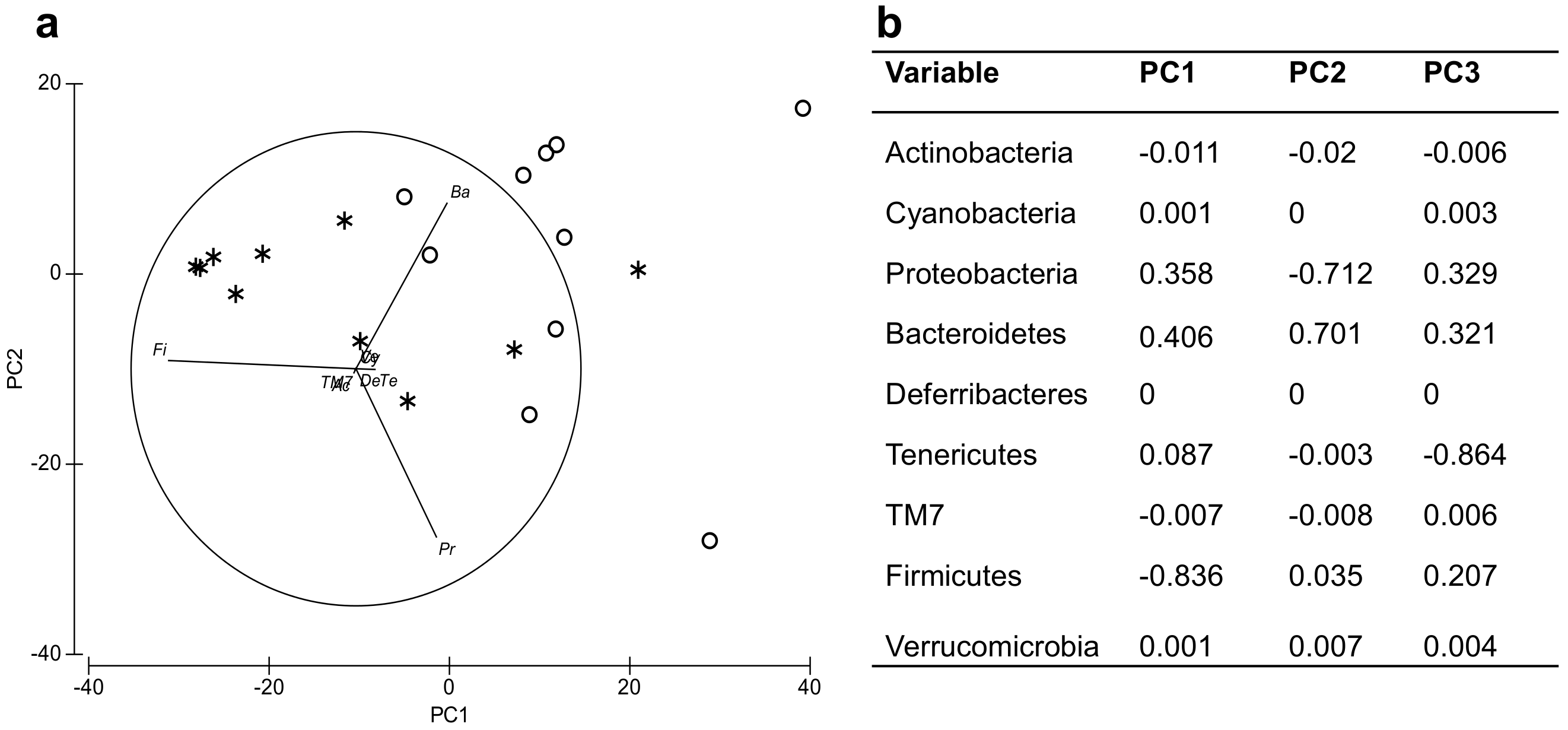

Supplement: S1 Fig — a) PC1 explained 71% of the variation, PC2 22.3% and PC3 6.5%. Star: chow diet; circle: high fat diet. b) Component loading derived from PCA analysis of bacterial phyla. (TIF) [file pone.0126931.s001.tif]

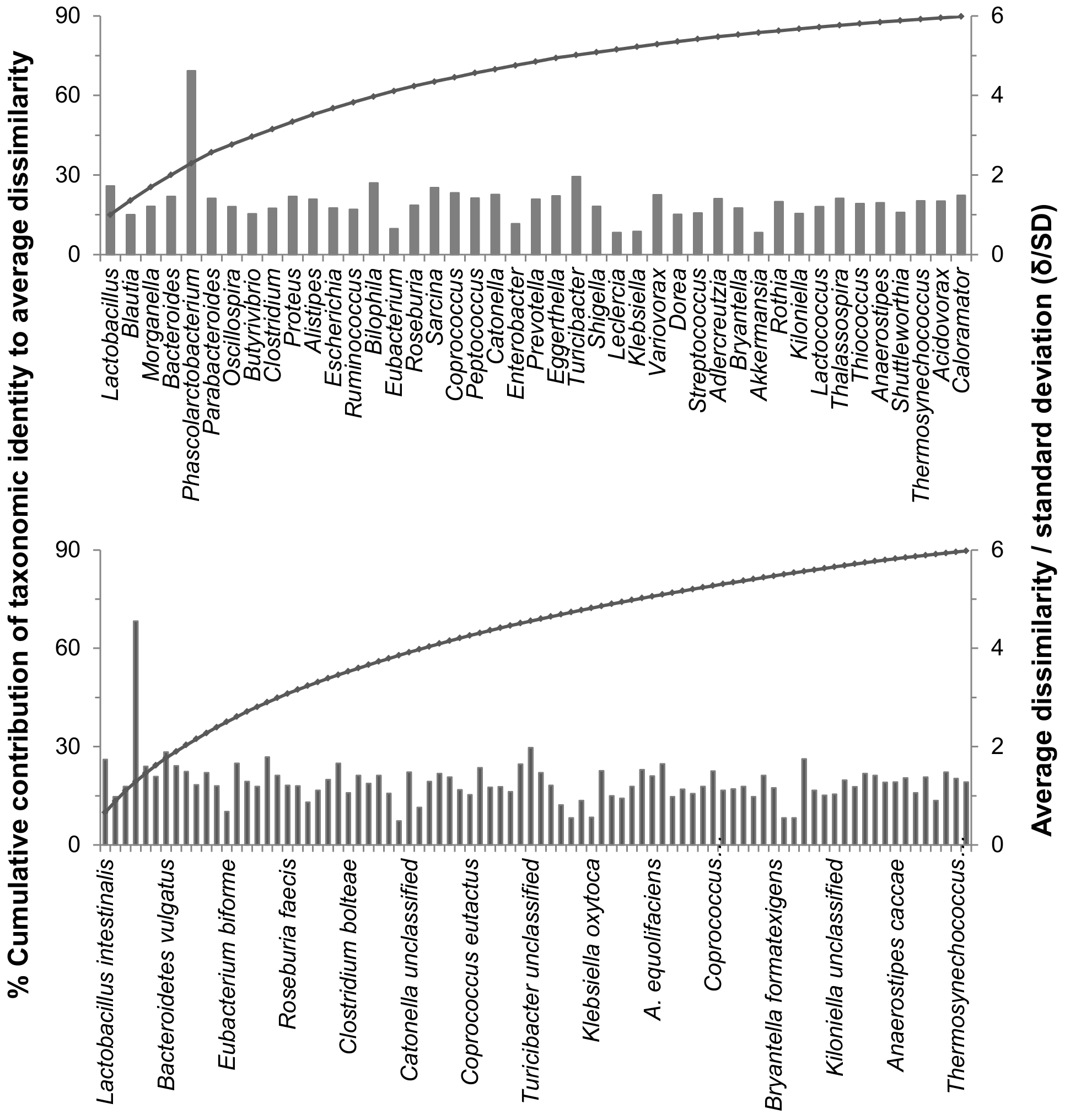

Supplement: S2 Fig — The primary axis displays the cumulative contribution that each taxonomic identity contributes to average dissimilarity between diet types. Upper limits of contributions displayed are 90% of average dissimilarity in genus and species. The secondary axis displays the average dissimilarity/standard deviation (δ/SD) of each taxonomic identity displayed. (TIF) [file pone.0126931.s002.tif]
